# Study on Lifestyle Evaluation Systems for the Health of Chinese Elderly

**DOI:** 10.3390/ijerph16020284

**Published:** 2019-01-21

**Authors:** Changliang Du

**Affiliations:** Department of Physical Education, Nanjing University of Aeronautics and Astronautics, Nanjing 210016, Jiangsu, China; duchangliang@nuaa.edu.cn

**Keywords:** Chinese elderly, lifestyle, evaluation system

## Abstract

*Background/Objective*: China is now faced with a serious population aging challenge, and the health of the Chinese elderly is becoming an imminent concern. Consequently, it is critical to establish a lifestyle evaluation system for promoting the health of the Chinese elderly. *Methods*: Interviews with experts and questionnaire surveys were conducted. Factor analysis, analytic hierarchy process, and statistical analyses were also adopted in this study. *Results*: Besides evaluation metrics and standards, a two-level category system including 50 indices and associated weights from three level 1 categories (physical and mental health and social wellbeing) and thirteen level 2 categories were obtained. *Discussion and Conclusions*: Based on the confirmatory factor analysis and Cronbach’s test, such an evaluation system excels in effectiveness and reliability, and is ready to be popularized in Chinese society. We expect that the Chinese elderly will benefit from our system and that it will lead to a healthy lifestyle accordingly.

## 1. Introduction

According to a demographics study, China is faced with an aging challenge, and the elderly population will be doubled in upcoming decades [1]. In order to overcome such challenges, from the government perspective, we need to, on the one hand, establish a senior care system in the foreseeable future; on the other hand, from the individual perspective, we need to research how to age healthily. The term “healthy aging” is often used as a synonym for “active aging” [2,3] or “successful aging” [4,5].

As defined by the WHO, healthy aging is “the process of developing and maintaining the functional ability that enables wellbeing in older age” [6]. Such a definition is established based on active aging, which is defined as “the process of optimizing opportunities for health, participation, and security in order to enhance the quality of life as people age” allowing people to “realize their potential for physical, social and mental wellbeing throughout the life course” [7].

Healthy aging can be influenced by numerous factors. Some of these factors can be controlled by individuals, such as lifestyle factors [3,8]. In 2008, the Chinese Ministry of Health carried out the “Health Literacy of Chinese Citizens—Basic Knowledge and Skills,” according to which a healthy lifestyle should include a balanced diet, moderate exercise, no smoking or drinking, and strong mental health [9]. The research group led by Dr. Walker proposed the concept of a health-promoting lifestyle, which is defined as spontaneous and multilevel behavior and the perception developed to maintain or promote individual health, and to obtain self-actualization and self-satisfaction. Such a lifestyle includes six dimensions: self-actualization, health responsibility, exercise, nutrition, interpersonal support, and stress management [10,11,12]. However, some social determinants including income and education are beyond the individual’s control. All in all, the healthy aging framework proposed by the WHO provides a useful model for understanding how social, personal, and behavioral determinants influence healthy aging. In our current work, we only focused on the aspect of lifestyle since it plays a critical role in healthy aging. Based on literature research, we found that in China there has been a shift in focus from passively studying how to cure diseases to aggressively researching ways to promote a healthy lifestyle and slow down the decline of physical function [13,14]. However, a systematic approach to evaluating the lifestyle of the Chinese elderly is lacking, and establishing such a lifestyle evaluation system will lay a foundation for a Chinese senior care system.

## 2. Materials and Methods

### 2.1. Hypotheses

Two hypotheses are formed in this study, as shown below:

- The health of the Chinese elderly can be evaluated based on indices from a two-level category system: three level 1 categories (physical and mental health and social wellbeing) and thirteen level 2 categories.
- Such an evaluation system excels in effectiveness and reliability and is ready to be popularized in Chinese society.

### 2.2. Objectives

The main objective of this work was to establish a lifestyle evaluation system for promoting the health of the Chinese elderly. The secondary objective was to validate the effectiveness and reliability of the proposed lifestyle evaluation system.

### 2.3. Subjects

In 2016, a questionnaire survey was conducted among 500 subjects from 15 community centers in the metropolitan area of Xi’an city through a combination of stratified and random samplings. The respondents are representative of the entire population, and the sample size and power of the study have been calculated. The questionnaire titled “Questionnaire on health-promoting lifestyles of the elderly from Xi’an” (see Appendix A) was designed to collect lifestyle data on the physical, mental, and social wellbeing perspectives. Among the 500 questionnaires, only 482 were considered to be valid with all questions answered. The subjects with valid answers were composed of 249 male and 233 females, out of which 170 subjects were with ages ranging from 60–69, 162 subjects from 70–79, and 150 subjects above 80. Then, we randomly divided those subjects into an exploratory factor analysis (EFA) group with 238 members and a confirmatory factor analysis (CFA) group with 244 members.

Two sociodemographic factors were considered during the survey: income and education level. However, the X_2_ test failed to show significant differences (*p* > 0.05) across different age groups (see Table 1). 

### 2.4. Methods

#### 2.4.1. WHO Framework

We researched the healthy aging framework proposed by the WHO [15] and selected the factors that are relevant to the lifestyle of the elderly.

#### 2.4.2. Interview with Experts and Questionnaire Survey

We interviewed seven experts within relevant fields on the health of the Chinese elderly and collected and analyzed the answers to deduce the indices obtained that are critical to a lifestyle evaluation system.

#### 2.4.3. Factor Analysis

After summarizing the feedback from the experts from Step 2.4.2, we screened indices based on statistical analysis and then used the remaining items to lay the foundation of our lifestyle evaluation system. In addition, we used the confirmatory factor analysis (CFA) method to evaluate the effectiveness of such a system.

#### 2.4.4. Analytic Hierarchy Process

We used the analytic hierarchy process (AHP) to determine the weights of selected indices obtained in Step 2.4.3. The AHP provides a comprehensive framework based on mathematics and psychology and is particularly effective in complex decision making.

#### 2.4.5. Determination of Weights

In order to obtain both the level 1 (by category) and level 2 (by individual) weights of indices, the analytic hierarchy process (AHP) was employed. All calculations were conducted in the MATLAB environment.

(1) Determining Level 1 Weights

(a) Build a Pairwise Comparison Matrix

We first built the following pairwise comparison matrix for the three criteria:

(1) $$A=\left[\begin{array}{ccc} 1 & 3 & 5\\ 1/3 & 1 & 3\\ 1/5 & 1/3 & 1 \end{array}\right],$$

(b) Compute the Weight Vector and the Largest Eigenvalue

We found the largest eigenvalue and associated eigenvector satisfying:

(2) $$\mathrm{AW}=\lambda_{max}W,$$

where $\lambda_{max}$ is the largest eigenvalue, and W is the associated normalized eigenvector. 

The results are shown below:

(3) $$W=\left[\begin{array}{ccc} 0.63 & 0.26 & 0.11 \end{array}\right],$$

(4) $$\lambda_{max}=3.0385,$$

(c) Calculate the Consistency Index

To measure the consistency of matrix A, we calculated its consistency index C.I.:

(5) $$C.I.=\frac{\lambda_{max}-n}{n-1},$$

If C.I.=0, we assume the matrix is totally consistent. The larger C.I. is, the less consistent the matrix becomes. To check whether the consistency is acceptable, we compared it with the appropriate one—random consistency index (R.C.I.) found in the standard table and calculated the consistency ratio (C.R.):

(6) $$C.R.=\frac{C.I.}{R.C.I.},$$

If C.R.<0.1, we assume that the consistency is acceptable; otherwise, we need to revise the subjective judgment represented by the matrix A. In this case, we had

(7) $$C.R.=\frac{\frac{\lambda_{max}-n}{n-1}}{R.I.}=\frac{0.019}{0.58}=0.033,$$

which suggests that the consistency of matrix A is satisfactory.

(2) Determining Level 2 Weights

Similarly, we obtained the largest eigenvalues, C.I., R.C.I., and C.R., which are summarized in Table 2.

From Table 2 we can see that all C.R. are below 0.1, suggesting that all comparison matrices have satisfactory consistencies. We used the normalized eigenvectors associated with the $\lambda_{max}$ as the level 2 weights, as shown in Table 3.

#### 2.4.6. Software Tools

In Step 3, the effectiveness test of the lifestyle evaluation system was conducted in AMOS 7.0 (SPSS Inc., Chicago, IL, USA). In Step 4, calculations involved in AHP were conducted in the MATLAB 7.0 (MathWorks Inc., Natick, MA, USA) environment. The remaining statistical calculation results in this study were obtained by SPSS 16.0 (SPSS Inc., Chicago, IL, USA).

## 3. Results

### 3.1. Establishment of a Lifestyle Evaluation System for the Chinese Elderly

#### 3.1.1. Preliminary Indices

As defined by the World Health Organization (WHO), health is a “state of complete physical, mental, and social wellbeing, and not merely the absence of disease or infirmity” [13]. Such a definition highlights that besides physical health, mental and social wellbeing states are also critical components of human health. As a result, our evaluation system will focus on evaluating lifestyles that might affect the health of the Chinese elderly from physical, mental, and social wellbeing aspects.

From the WHO framework where indices for evaluating each aspect of human health have been proposed and validated, we have summarized all available results and added more indices based on our interview results with experts (professors and doctors) who have rich knowledge and experience in the health of the Chinese elderly. Finally, we concluded our preliminary research with seventy-two indices in total from three aspects of health: physical, mental, and social wellbeing.

#### 3.1.2. Indices Screening Based on Questionnaire Survey

We designed a questionnaire on the level of importance of each index based on the Likert Scale from 1 (not important at all) to 5 (very important). We asked the same experts to complete the questionnaires, and then we collected the answers and conducted the statistical analysis. We calculated the mean $\bar{x}$ and coefficient of variance (CV) for each index, kept indices with $\bar{x}\geq 4$ and CV<0.3 in our evaluation system, and excluded those that did not meet such criteria. Finally, we had sixty-two indices in our system for evaluating the health of the Chinese elderly, in which thirty indices were on physical health, 18 on mental health, and 14 on social wellbeing.

#### 3.1.3. Indices Screening Based on Factor Analysis

First, to find out whether the data were suitable for factor analysis, we conducted the Kaiser–Meyer–Olkin (KMO) test and Bartlett’s test. If the KMO test gave a value larger than 0.6 and the *p*-value of the Bartlett’s test was less than 0.05, we could assume that both tests demonstrated statistical significance, i.e., the data would be suitable for factor analysis. 

Then, we further screened the indices based on factor analysis and removed the index if (a) its communality was low; (b) it showed in only one factor; and (c) it gave high factor loadings in more than one factor. We iteratively conducted principal component analysis and varimax rotation and removed indices until no such criteria were met. Results are summarized below.

(1) Factor Analysis on Indices Impacting Physical Health

While the KMO test gave 0.81, the *p*-value of Bartlett’s test was less than 0.05. As a result, both tests demonstrated statistical significance, i.e., the factor analysis on the data is statistically valid. 

Six factors with eigenvalues above 1 were selected after the analysis, and twenty-six indices were included in total and explained 60.26% of total variance contained in the dataset. Such factors were composed of 3, 5, 4, 4, 6, and 4 indices reflecting the physical condition, eating routine, exercise habits, quality of sleep, use of drugs or alcohol, and living environment, respectively.

(2) Factor Analysis on Indices Impacting Mental Health

The KMO test and Bartlett’s test gave 0.76 and *p* < 0.05, respectively, showing that the data were suitable for factor analysis. Four factors with eigenvalues above 1 were selected, thirteen indices were included in total, and they explained 56.85% of total variance. The factors contained 4, 3, 3, and 3 indices related to the mental status, emotion control, stress handling, and self-regulation, respectively.

(3) Factor Analysis on Indices Impacting Social Well Being

Similarly, the factor analysis on indices data impacting social wellbeing was also proven to be statistically justifiable, as demonstrated by the KMO and Bartlett’s tests giving 0.88 and *p* < 0.05. In this case, we chose three factors with eigenvalues above 1, eleven indices were included, and they explained 52.66% of total variance. The three factors were composed of 5, 3, and 3 indices regarding prosocial behaviors, interpersonal relationships, and social recognition, respectively.

#### 3.1.4. Final Indices

Table 3 summarizes the final indices and associated weights used for evaluating the lifestyle of the Chinese elderly. The proposed two-level category system includes 50 indices from three level 1 categories (physical and mental health and social wellbeing) and thirteen level 2 categories. The physical health category can be further divided into physical condition, eating routine, exercise habits, quality of sleep, use of drugs or alcohol, and living environment. While mental health includes mental status, emotion control, stress handling, and self-regulation, the social wellbeing class consists of prosocial behaviors, interpersonal relationships, and social recognition.

### 3.2. Test of Lifestyle Evaluation System for the Chinese Elderly

#### 3.2.1. Validity Test

As shown in Section 3.1, the content validity of the evaluation indices has been corroborated by both experts and statistical analysis. To further test the structure validity of the lifestyle evaluation system, we conducted the confirmatory factor analysis (CFA). The data we used were collected from a testing group of 244 members randomly selected from 500 subjects, and the results are summarized in Table 4. From the table we can see that it has satisfactory goodness of fit, suggesting the model has decent structure validity.

In addition, to study the generalizability of the established lifestyle evaluation system, we tested its external validity. It is universally acknowledged that lifestyle plays a fundamental role in promoting individual health. As a result, we correlated the individual performance of our lifestyle evaluation system with that of the health self-evaluation system published by WHO [16] and obtained r = 0.78 (*p* < 0.01). Such a result suggests that our lifestyle evaluation system has decent generalizability, i.e., external validity.

#### 3.2.2. Reliability Test

Table 5 summarizes the assessment results of reliability based on Cronbach’s test. We can see that Cronbach’s alphas of physical health, mental health, social wellbeing, and overall equal 0.82, 0.79, 0.78, and 0.86, respectively. Such a high value of alpha indicates that our lifestyle evaluation system is capable of producing stable and consistent results.

## 4. Discussion

After finalizing the indices used for evaluating the lifestyle of the Chinese elderly, we established assessment criteria based on (1) current standards, state policies, and regulations, including “Chinese Resident Health Literacy”, “Chinese Dietary Guidelines”, and “National Fitness Program and Regulations”; (2) established scientific theories and state-of-the-art research regarding human health and promoting healthy lifestyles. For example, our assessment criteria for the eating routine is “with a regular and balanced diversity of sources of protein, fiber, carbohydrate, lipid, vitamins, and minerals”. We expected the respondents to assess their individual performance based on the Likert Scale from 1 (nonconformity) to 5 (conformity).

The results from both the validity and reliability tests show that our lifestyle evaluation system excels in structure validity, generalizability, stability, and consistency. In our study, a new system was established to evaluate from different aspects the lifestyles of the Chinese elderly. This survey is more comprehensive than the one established by the WHO. Although the subjects in this paper were from Xi’an city only, we are confident that subjects from other cities will generate similar results. In the future, we will test such a system on subjects from other cities such as Beijing and Shanghai.

However, there are a few limitations in this study. First, the data used in our study were collected through filling out questionnaires, and currently there is a lack of objective measuring tools that are shielded from subjective views. In the future, we will incorporate indices obtained from physiological experiments in the evaluation system. Second, our current cross-sectional study has not validated the effects of intervention. As a result, in the future, we will design intervention strategies for lifestyle improvements of the elderly and conduct associated tests. 

## 5. Conclusions

In the current study, we established a novel lifestyle evaluation system for the Chinese elderly and obtained associated indices and relevant weights from reviewing the relevant WHO framework, interviews with experts, questionnaire surveys, factory analysis, and an analytic hierarchy process. The proposed two-level category system includes 50 indices from three level 1 categories (physical and mental health and social wellbeing) and thirteen level 2 categories. The validity and reliability of such a lifestyle evaluation system have been verified through confirmatory factor analysis (CFA) and Cronbach’s test. As a result, the system is ready to be tested in more cities and is expected to be promoted in Chinese society during the next stage. Furthermore, it can contribute to the Chinese senior care system in the future.

## Figures and Tables

**Table 1:** Sample distribution based on sociodemographic factors.

Factors	Level	Age ≤ 69	Age 70–79	Age ≥ 80
Income	Low	17	12	9
	Mid-class	120	110	105
	Wealthy	43	40	36
Education	High school or less	28	42	75
	Bachelor’s degree	117	99	75
	Master’s degree or higher	25	21	10

**Table 2:** Indices related to level 2 weights and consistency test.

Factor	$\lambda_{\max}$	C.I.	R.C.I.	C.R.
*B*_1_	6.18	0.036	1.24	0.029
*B*_2_	4.10	0.004	0.90	0.004
*B*_3_	3.02	0.012	0.58	0.021

C.I. = consistency index; R.C.I. = random consistency index; C.R. = consistency ratio.

**Table 3:** Indices and weights for evaluating the lifestyle of the Chinese elderly.

Level 1 Category	Level 1 Weights	Level 2 Category	Level 2 Weights	Combined Weights	Number of Indices
Physical health	0.63	Physical condition	0.41	0.26	3
		Eating routine	0.19	0.12	5
		Exercise habits	0.19	0.12	4
		Quality of sleep	0.11	0.07	4
		Use of drugs or alcohol	0.06	0.04	6
		Living environment	0.04	0.03	4
Mental health	0.26	Mental status	0.35	0.09	4
		Emotion control	0.35	0.09	3
		Stress handling	0.19	0.05	3
		Self-regulation	0.11	0.03	3
Social wellbeing	0.11	Prosocial behaviors	0.33	0.04	5
		Interpersonal relationships (including family)	0.57	0.06	3
		Social recognition	0.10	0.01	3

**Table 4:** Confirmatory factor analysis (CFA) results of the proposed lifestyle evaluation system.

χ^2^	df	χ^2^/df	CFI	NNFI	IFI	RMSEA	AIC
29.85	9	3.32	0.95	0.93	0.96	0.06	10.12

CFI = comparative fit index; NNFI = non-normed fit index; IFI = incremental fit index; RMSEA = root mean square error of approximation; AIC = Akaike’s information criterion.

**Table 5:** Reliability test results.

Category	Index Number	Cronbach’s α
Physical health	26	0.82
Mental health	13	0.79
Social well being	11	0.78
Overall	50	0.86

## Abbreviations

AHP	analytic hierarchy process
AIC	Akaike’s information criterion (AIC)
CFA	confirmatory factor analysis
C.I.	consistency index
C.R.	consistency ratio
CFI	comparative fit index
IFI	incremental fit index
KMO test	Kaiser-Meyer-Olkin test
NNFI	non-normed fit index
RMSEA	root mean square error of approximation
R.C.I.	random consistency index
WHO	world health organization

## Appendix A. Questionnaire on Health-Promoting Lifestyles of the Elderly from Xi’an

Thanks for participating in the survey! This questionnaire is only for understanding of the current lifestyles of the elderly and contributory factors. The results will be used only for policy making of the government in order to tackle the aging challenges and improve social welfare programs. This questionnaire survey is absolutely anonymous and all information collected will stay confidential.

1. Do you smoke? (1) Never (jump to Q3) (2) Used to, not now (3) Yes
2. How many cigarettes do you smoke every day? How old were you when you started/quitted smoking?
3. How often do you drink? (1) Never (jump to Q5) (2) Occasionally (once/twice per week) (3) Often (around 3 to 6 times per week) (4) Daily (5) More than once per day
4. How much do you drink each time? Do you drink wine, beer, or liquor? How old were you when you started/ quitted drinking? Have you been drunk in the last month?
5. How long do you sleep every night? (1) t < 6 hours (2) 6 hours ≤ t < 7 hours (3) 7 hours ≤ t < 8 hours (4) t ≥ 8 hours (5) Irregular
6. When do you fall sleep? (1) Before 20:00 (2) 20:00–22:00 (3) 22:01–24:00 (4) After 24:00 (5) Irregular
7. How often do you exercise per week? (1) Never (2) Once/twice (3) 3–6 times (4) Daily (5) More than once per day
8. How often do you have a physical exam? (1) Never (jump to Q10) (2) Irregular (3) Every couple of years (4) Annually
9. I have a physical exam because (1) Company recommendation (2) Community recommendation (3) Personal habit (4) Physical discomfort
10. Do you regularly eat three meals every day? (1) Yes (2) Occasionally No (3) Never
11. Do you care about a healthy diet? (1) Very much (2) To some extent (3) Never
12. How often do you participate in social events? (1) Frequently (2) Occasionally (3) Rarely (4) Never
13. Do you stay close with your children? (1) Yes (2) To some extent (3) No (4) No children
14. Do you stay close with your spouse? (1) Yes (2) To some extent (3) No (4) No spouse.
